# IL-36α and Lipopolysaccharide Cooperatively Induce Autophagy by Triggering Pro-Autophagic Biased Signaling

**DOI:** 10.3390/biomedicines9111541

**Published:** 2021-10-26

**Authors:** Zaid I. I. Al-Luhaibi, Áron Dernovics, György Seprényi, Ferhan Ayaydin, Zsolt Boldogkői, Zoltán Veréb, Klára Megyeri

**Affiliations:** 1 Department of Medical Microbiology, Albert Szent-Györgyi Medical School, University of Szeged, Dóm tér 10, H-6720 Szeged, Hungary; alluhaibi.zaid@med.u-szeged.hu (Z.I.I.A.-L.); dernovics.aron@med.u-szeged.hu (Á.D.); 2 Department of Anatomy, Histology and Embryology, Albert Szent-Györgyi Medical School, University of Szeged, Kossuth L. sgt. 40, H-6724 Szeged, Hungary; seprenyi.gyorgy@med.u-szeged.hu; 3 Hungarian Centre of Excellence for Molecular Medicine (HCEMM) Nonprofit Ltd., Római krt. 21, H-6723 Szeged, Hungary; ferhan.ayaydin@hcemm.eu; 4 Biological Research Centre, Laboratory of Cellular Imaging, Eötvös Loránd Research Network, Temesvári krt. 62, H-6726 Szeged, Hungary; 5 Department of Medical Biology, Albert Szent-Györgyi Medical School, University of Szeged, Somogyi Béla u. 4, H-6720 Szeged, Hungary; boldogkoi.zsolt@med.u-szeged.hu; 6 Regenerative Medicine and Cellular Pharmacology Laboratory, Albert Szent-Györgyi Medical School, University of Szeged, Korányi fasor 6, H-6720 Szeged, Hungary; vereb.zoltan@med.u-szeged.hu

**Keywords:** IL-36α, LPS, autophagy, LC3B, Beclin-1

## Abstract

Autophagy is an intracellular catabolic process that controls infections both directly and indirectly via its multifaceted effects on the innate and adaptive immune responses. It has been reported that LPS stimulates this cellular process, whereas the effect of IL-36α on autophagy remains largely unknown. We therefore investigated how IL-36α modulates the endogenous and LPS-induced autophagy in THP-1 cells. The levels of LC3B-II and autophagic flux were determined by Western blotting. The intracellular localization of LC3B was measured by immunofluorescence assay. The activation levels of signaling pathways implicated in autophagy regulation were evaluated by using a phosphokinase array. Our results showed that combined IL-36α and LPS treatment cooperatively increased the levels of LC3B-II and Beclin-1, stimulated the autophagic flux, facilitated intracellular redistribution of LC3B, and increased the average number of autophagosomes per cell. The IL36α/LPS combined treatment increased phosphorylation of STAT5a/b, had minimal effect on the Akt/PRAS40/mTOR pathway, and reduced the levels of phospho-Yes, phospho-FAK, and phospho-WNK1. Thus, this cytokine/PAMP combination triggers pro-autophagic biased signaling by several mechanisms and thus cooperatively stimulates the autophagic cascade. An increased autophagic activity of innate immune cells simultaneously exposed to IL-36α and LPS may play an important role in the pathogenesis of Gram-negative bacterial infections.

## 1. Introduction

**Figure 1:**
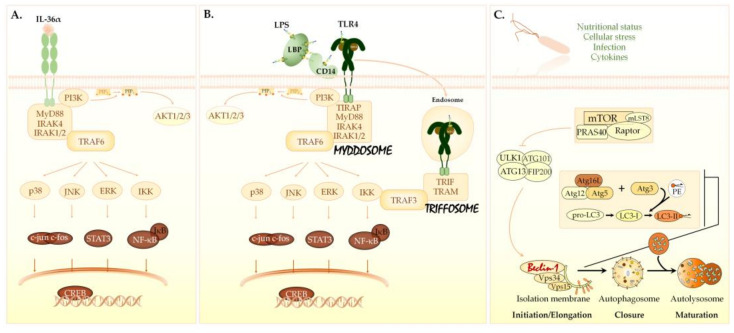
Signaling pathways triggered by IL-36α, LPS, and autophagy inducers. (**A**) The IL-36α signaling pathway. IL-36α/β/γ bind to the IL-36R (IL-1Rrp2) and use the IL-1 receptor accessory protein (IL-1RAcP) as a co-receptor. Following ligand binding, the TIR domain—located in the intracellular portion of the IL-36R:IL-1RAcP heterodimer—recruits MyD88 adaptor protein, which in turn interacts with IRAKs and TRAF-6. The MyD88/IRAK/TRAF6 platform activates AP-1, CREB and NF-κB transcription factors via IKK, ERKs, JNKs, and p38. (**B**) The LPS/TLR4 signaling pathway. LBP and CD14 bind and transfer LPS monomers to the TLR4/MD-2 heterodimer. The dimerized TIR domain in the cytoplasmic portion of TLR4 consecutively recruits TIRAP, MyD88, and IRAK proteins, thus leading to the assembly of a supramolecular complex termed the myddosome. The LPS-TLR4/MD2 complexes can also be internalized into endosomes. The TIR domain of endosomal TLR4 binds TRAM, which attracts TRIF resulting in the formation of another complex called the triffosome. Myddosomes and triffosomes recruit TRAF6 or TRAF3 and activate AP-1, NF-κB, CREB, and IRF-3 via MAPKs, IKK, and PI3K. (**C**) The autophagy-related signaling pathway. The major element in the regulation of autophagy is the mTORC1. Besides mTOR, mTORC1 is comprised of the Raptor and mLST8 molecules. The non-core components associated with mTORC1 include PRAS40 and DEPTOR. Autophagy inducers inhibit mTORC1, and thereby activate the autophagic cascade. Inhibition of mTORC1 results in the consecutive activation of the ULK1 and PI3KC3 complexes. The PI3KC3 complex contains Beclin-1 protein. The activated PI3KC3 induces local production of PI3P at the endoplasmic reticulum membrane and thereby promotes the generation of an omegasome from which the isolation membrane is generated. PI3P recruits two ubiquitin-like conjugation systems, the ATG12–ATG5-ATG16L1 and ATG7/ATG3 complexes, to the omegasome. ATG3 forms a covalent bond between the membrane-resident phosphatidylethanolamine and the microtubule-associated protein 1 light chain 3B-I (LC3B-I) protein. The ATG12–ATG5-ATG16L1 complex enhances the ATG3-mediated conjugation of LC3B-I. The lipidated LC3B protein is termed LC3B-II. LC3B-II is essential for the elongation and closure of the phagophore membrane, trafficking of the autophagosomes, and their fusion with lysosomes. AP-1, activator protein 1; CREB, cAMP response element-binding protein; DEPTOR, DEP-domain containing mTOR-interacting protein; ERKs, extracellular signal-regulated kinases; IKK, IκB kinase; IRAKs, IL-1 receptor-associated kinases; IRF-3, interferon regulatory factor 3; JNKs, c-Jun N-terminal kinases; LBP, LPS-binding protein; MD-2, myeloid differentiation factor 2; mLST8, mLST8; mammalian lethal with sec-13 protein 8; mTOR, mechanistic target of rapamycin; mTORC1, mTOR complex 1; MyD88, myeloid differentiation primary response 88; NF-κB, nuclear factor-κB; PE, phosphatidylethanolamine; PI3K, class I phosphatidylinositol 3-kinase; PI3KC3, class III phosphatidylinositol 3-kinase; PI3P, phosphatidylinositol-3-phosphate; PRAS40, proline-rich Akt substrate of 40 kDa; Raptor, regulatory-associated protein of mTOR; TIR, Toll/IL-1 receptor domain; TLR4, Toll-like receptor 4; TRAF-6, tumor necrosis factor receptor-associated kinase 6; TRAM, TRIF-related adaptor molecule; TRIF; TIR domain-containing adaptor-inducing interferon-β; ULK1, Unc-51-like kinase 1; WIPI, WD repeat domain phosphoinositide-interacting proteins.

Interleukin-36α (IL-36α), IL-36β, IL-36γ, and IL-36 receptor antagonist (IL-36Ra) belong to the IL-36 subfamily of the IL-1 cytokine family [1,2,3]. The IL-36 subfamily includes three agonist cytokines (IL-36α/β/γ) as well as the natural antagonist of IL-36 (IL-36Ra). Expression of IL-36α can be observed at low levels in many different tissues, most notably in the skin, esophagus, tonsil, lung, gut, and brain. IL-36α can also be secreted by the immune cells including monocytes/macrophages and T cells [4]. IL-36α/β/γ are highly induced in response to several stimuli including cytokines, Toll-like receptor agonists, bacteria, viruses, and various pathological conditions. The IL-36 subtypes are synthesized as precursor proteins. Cleavage of the precursor form of IL-36α at a specific site located at nine amino acids N-terminal to a conserved A-X-Asp motif highly increases the affinity of the truncated cytokine to the receptor and enhances its biological activity [5,6,7]. The truncated IL-36α/β/γ bind to the same heterodimeric receptor complex. Ligand binding leads to the sequential formation of a signaling complex containing some adaptors, kinases, and the E3 ubiquitin ligase, tumor necrosis factor receptor-associated kinase 6 (TRAF-6). This platform triggers a signaling cascade involving mitogen-activated protein kinases (MAPKs) and the activation of several transcription factors (Figure 1A) [3,7,8,9,10].

IL-36 subtypes stimulate the production of several cytokines (IL-1α, IL-1β, IL-2, IL-4, IL-6, IL-8, IL-10, IL-12, IL-17, IL-18, IL-22, IL-23, TNFα, HB-EGF, and IFN-γ), colony-stimulating factors (GM-CSF and G-CSF), chemokines (CCL1-3, CCL20, CXCL1-3, CXCL5, CXCL10, CXCL12), and cell adhesion molecules (VCAM-1, ICAM-1) in various cell types [11,12,13]. Furthermore, IL-36 cytokines increase the intracellular level of antimicrobial peptides (beta-defensins 2 and 3, LL37, and protein S100-A7) and elevate the expression of major histocompatibility complex class 2 and clusters of differentiation 14 (CD14), CD40, CD80/CD86, and CD83 [12,13,14]. IL-36α/β/γ thereby activate innate immune cells and induce inflammation. The pro-inflammatory IL-36 subfamily members also modulate the adaptive immune responses by stimulating TH-cell proliferation and promoting CD4+ T lymphocyte differentiation toward TH1, TH17, and TH9 phenotypes. IL-36γ was shown to activate natural regulatory T-cells (Tregs) [15] and inhibit the generation of induced Tregs [16]. In acute resolving inflammation, IL-36 α/β/γ has been suggested to facilitate the elimination of pathogenic microorganisms, the resolution of tissue injury, and the restoration of tissue integrity. However, in chronic inflammatory processes, these IL-36 subtypes can exert a pathogenic effect by amplifying inflammatory processes [17,18,19].

Lipopolysaccharide (LPS) is a powerful immunomodulatory molecule that contributes to the pathogenesis and clinical symptoms of infections caused by Gram-negative bacteria. LPS is the major structural component of the outer bacterial membrane and is composed of O antigen, poly/oligosaccharide core, and lipid A, termed endotoxin [20,21]. LPS is a pathogen-associated molecular pattern (PAMP) detected by sensor molecules located in the cytoplasmic and endosomal membranes as well as in the cytoplasm of cells [22,23,24]. Extracellular LPS is sensed and extracted from the bacterial outer membrane by the LPS-binding protein (LBP) and CD14 [22,23,24]. CD14 transfers monomeric LPS molecules to the Toll-like receptor 4 (TLR4) leading to the assembly of a supramolecular complex termed the myddosome. The LPS-TLR4 complexes can also be internalized into endosomes resulting in the formation of another complex called the triffosome. These platforms trigger a signaling cascade involving MAPKs and the activation of several transcription factors (Figure 1B) [20,22,23,24,25]. The activated transcription factors turn on the expression of various cellular genes encoding inflammatory mediators including cytokines [20,22,23]. In addition to TLR4, another group of cytoplasmic membrane receptors—the transient receptor potential (TRP) cationic channels—can bind LPS [20,26]. TRP cation channel subfamily V members 2 and 4 (TRPV2 and TRPV4) and TRPM7 also contribute to the LPS-mediated activation of innate immune cells by triggering intracellular Ca2+ mobilization and secretion of nitric oxide [27,28]. Additionally, intracellular LPS within the cytoplasm of cells directly binds to the caspase activation and recruitment domains of caspase-4/5 in humans and caspase-11 in mice, which in turn leads to activation of inflammatory caspases, secretion of IL-1β and IL-18, as well as induction of pyroptosis [29]. In localized infections caused by Gram-negative bacteria, LPS-mediated activation of the immune response is protective by restricting bacterial invasion whereas the exaggerated inflammation seen in systemic infections is of pivotal pathogenetic and prognostic importance.

Autophagy is an intracellular metabolic process in which cytoplasmic target molecules are transferred to the lysosome for degradation and recycling [30,31,32]. Depending on the type of cargo delivery, three different forms of autophagy can be distinguished: (1) macroautophagy, (2) microautophagy, and (3) chaperon-mediated autophagy [32]. The difference among these types is how the constituents to be degraded are transported to the lysosome. Macroautophagy (hereafter referred to as autophagy) occurs under basal conditions and can be stimulated by environmental cues, nutrient starvation, growth factor depletion, various pathological conditions, infections, hypoxia, or pharmacological treatment [33]. The major element in the regulation of autophagy is the mechanistic target of rapamycin complex 1 (mTORC1) [34,35]. Autophagy inducers inhibit mTORC1, and thereby activate the autophagic cascade. Inhibition of mTORC1 results in the activation of the class III phosphatidylinositol 3-kinase (PI3KC3) complex. The PI3KC3 complex contains Beclin-1 protein [36,37]. The activated PI3KC3 induces local production of phosphatidylinositol-3-phosphate (PI3P) at the endoplasmic reticulum membrane and thereby promotes the generation of an omegasome from which the isolation membrane is generated [37,38,39]. PI3P recruits two ubiquitin-like conjugation systems to the omegasome, which form a covalent bond between the membrane-resident phosphatidylethanolamine (PE) and the microtubule-associated protein 1 light chain 3B-I (LC3B-I) protein [38,39]. The lipidated LC3B, termed LC3B-II, is essential for the elongation and closure of the phagophore membrane, trafficking of the autophagosomes, and their fusion with lysosomes [40,41,42]. The level of LC3B-II reflects the cellular autophagic activity and it is used as a marker to measure autophagy. Upon autophagy induction, LC3B translocates from the cytoplasm to autophagic membranes, and can be detected as fluorescent puncta (Figure 1C) [41].

The autophagosomes eventually fuse with lysosomes, and the content of the autophagic cargo is thus degraded and made available for reuse [43,44]. Autophagy is essential for the maintenance of cellular homeostasis and plays a substantial role in pathological processes including bacterial infections [33,45]. Autophagic capture and delivery of bacteria to lysosomes functions as a protective cellular antimicrobial defense mechanism known as xenophagy [46]. Autophagy also controls bacterial infections indirectly via its multifaceted effects on the innate and adaptive immune responses [47,48]. mTORC1 signaling affects the maturation, metabolic activity, activation, and differentiation of innate immune cells [49,50]. The mTORC1 pathway can also stimulate the production of type I interferons (IFN), IL-10, and transforming growth factor-β, and downregulates the expression of IL-6, IL-12, IL-23, and TNF-α in monocytes, macrophages, and dendritic cells [51]. The autophagic machinery promotes the secretion of IL-1β, IL-18, and high-mobility group protein B1 and also facilitates antigen presentation to CD4^+^ and CD8^+^ T-cells. Conversely, immune processes may profoundly alter the cellular autophagic activity [47]. Ample evidence indicates that LPS induces autophagy via some mTOR-independent mechanisms. In response to binding of LPS to TLR4, the myddosomes and triffosomes recruit, whereas TRAF6 ubiquitinates Beclin-1, leading to the more efficient assembly of PI3KC3 with subsequent activation of the autophagic cascade [52,53].

Besides the signaling events elicited by pathogen-recognition receptors, cytokines may also modulate autophagy. IL-1α, IL-1β, IL-2, IL-6, IL-17, TNF-α, TWEAK (tumor necrosis factor-like weak inducer of apoptosis), and IFN-γ act as inducers whereas IL-4, IL-10, and IL-13 function as inhibitors of autophagy [54,55]. Recent data demonstrate the pro-autophagic effect of IL-36β and IL-36γ [56,57]. IL-36β enhanced the expression of key autophagy markers, LC3-II, Beclin-1, and p62, as well as elevated autophagic flux in CD4^+^CD25^+^ Treg cells [57]. This IL-36 subtype played protective role in sepsis by diminishing the immunosuppressive activity of CD4^+^CD25^+^ Tregs [57]. The signal transduction mechanisms involved in IL-36β-mediated induction of autophagy, however, have not been identified. IL-36γ triggered autophagy via WNT5A-induced noncanonical WNT signaling in macrophages infected with *Mycobacterium tuberculosis* [56]. Moreover, IL-36γ-induced autophagy promoted the killing of intracellular bacteria [56]. In contrast, the effect of IL-36α on the autophagic process has not yet been elucidated. Infections involve a variety of pathogen-related molecular pattern (PAMP)—cytokine recognition events that can profoundly affect the antimicrobial response of immune cells. Thus, we investigated the effect of IL-36α upon endogenous and LPS-induced autophagy. We report that IL-36α can synergize with LPS for the induction of the autophagic process in the THP-1 cell line by triggering pro-autophagic biased signaling.

## 2. Materials and Methods

### 2.1. Chemical Compounds

Human recombinant IL-36α (Biomol GmbH, Hamburg, Germany) was prepared in sterile distilled water and used at 10 ng/mL concentration in all experiments. Human recombinant IL-36Ra (Sigma–Aldrich, St. Louis, MO, USA) was prepared in sterile distilled water and used at 20-fold molar excess.

A stock solution of autophagy inhibitor bafilomycin A1 BFLA (Santa Cruz Biotechnology, Dallas, TX, USA) was prepared in dimethyl sulfoxide. BFLA was used at a concentration of 100 nM in all experiments.

### 2.2. Cell Culture

The THP-1 human pro-monocytic cell line was grown in Dulbecco’s modified Eagle’s minimal essential medium (Sigma–Aldrich) supplemented with 10% fetal calf serum (Lonza, Verviers, Belgium) and 1% of an antibiotic/antimycotic (AB/AM) solution (Lonza) at 37 °C in a 5% CO_2_ atmosphere.

### 2.3. Immunofluorescence Assay

Cytospin cell preparations were fixed in methanol acetone (1:1) for 10 min at −20 °C. The cells were treated with 1% bovine serum albumin in PBS for 30 min at 37 °C to block non-specific binding of the antibodies. To detect LC3B, the slides were stained with a 1:150 dilution of rabbit polyclonal antibody to LC3B (Sigma-Aldrich) for 1 h at 37 °C. To detect Beclin-1, the slides were stained with a 1:100 dilution of rabbit polyclonal antibody to Beclin-1 (Sigma-Aldrich) for 1 h at 37 °C. After washing with PBS, the samples were reacted with a 1:300 dilution of CF488A-conjugated anti-rabbit antibody (Sigma-Aldrich) for 1 h at 37 °C. The cells were visualized by confocal microscopy using an Olympus FV1000 confocal laser scanning microscope using UPLSAPO 60X (N.A. 1.35) oil immersion objective and 488 nm laser excitation with 500–600 nm detection range. LC3B-positive vacuoles were automatically quantified for each field after subtraction of the background level and establishment of an intensity threshold using Image J software (U.S. National Institutes of Health, Bethesda, MD, USA). The numbers of the LC3B-positive puncta were normalized by the numbers of cells in each field. An average of 500 cells was analyzed for each condition. The fluorescence intensity of LC3B was determined using the surface plot functions of the Image J software. The mean fluorescence intensity (MFI) method was used to quantify the fluorescent signal intensities of cells. ImageJ software was used to draw an outline around each cell, and the MFI was measured. The corrected total cell fluorescence (CTCF) was calculated via the following formula: CTCF = integrated density—(area of selected cell × mean fluorescence of background readings).

### 2.4. Western Blot Assays

The cells were homogenized in CytoBuster lysis buffer (Merck KGaA, Darmstadt, Germany), and the mixture was then centrifuged at 10,000 g for 10 min to remove cell debris. Protein concentrations of cell lysates were determined using the Bio-Rad protein assay (Bio-Rad Laboratories Inc., Hercules, CA, USA). Supernatants were mixed with Laemmli sample buffer and boiled for 3 min. Aliquots of the supernatants were resolved by SDS-PAGE and electrotransferred onto Immun-Blot polyvinylidene difluoride (PVDF) membranes (Bio-Rad Laboratories Inc.). The membranes were blocked in PBS containing 0.05% Tween 20, and 5% dried non-fat milk (Difco Laboratories Inc., Detroit, MI, USA). The pre-blocked blots were probed with the appropriate antibodies for 4 h in PBS containing 0.05% Tween 20, 1% dried non-fat milk and 1% bovine serum albumin (Sigma-Aldrich). Rabbit anti-LC3B (Sigma-Aldrich) and rabbit anti-β-actin (Sigma-Aldrich) primary antibodies were used at a 1:1000 dilution. Blots were then incubated for 2 h with peroxidase-conjugated anti-rabbit antibody (Sigma-Aldrich). Membranes were developed using a chemiluminescence detection system (GE Healthcare, Chicago, IL, USA). The blots were scanned and the relative band intensities were quantified using ImageJ software (U.S. National Institutes of Health, Bethesda, MD, USA) [58].

### 2.5. Phospho-Kinase Array Analysis

A human phospho-kinase array (R & D Systems Inc., Minneapolis, MN, USA) was used to measure the relative phosphorylation levels of 43 signaling molecules. Control cells and cultures treated with IL-36α and LPS alone or in combination for 30 min were homogenized in a lysis buffer and centrifuged for five min at 14,000× *g*. Protein concentrations of the supernatants were determined using a Bio-Rad protein assay (Bio-Rad Laboratories Inc.). From each treatment group, three individual samples containing 100–100 µg protein were combined. After blocking, 300 μg of protein was incubated overnight at 4 °C with each array comprising two technical replicates. After washing, the arrays were reacted with a cocktail of phospho-site-specific biotinylated antibodies for two hours at room temperature, carefully washed again, and incubated with streptavidin–peroxidase for 30 min at room temperature. Signals were developed using a chemiluminescence detection system. The arrays were scanned, spot densities of phospho-proteins were quantified using ImageJ software (U.S. National Institutes of Health, Bethesda, MD, USA) [58] and normalized to those of positive controls on the same membrane after subtraction of background values.

### 2.6. Statistical Analysis

Statistical significance was analyzed by one-way ANOVA followed by Tukey’s or Sidak’s multiple comparison post-hoc tests. All statistical analyses were performed using GraphPad Prism 6 software (GraphPad Software Inc., San Diego, CA, USA), and *p* values less than 0.05 were considered statistically significant.

## 3. Results

### 3.1. The Effects of IL-36α and LPS on the Subcellular Localization of LC3B in the THP-1 Cell Line

To elucidate how IL-36α and LPS affect the basal autophagy, we treated THP-1 cells with IL-36α and LPS alone or in combination and measured the subcellular localization of LC3B.

**Figure 2:**
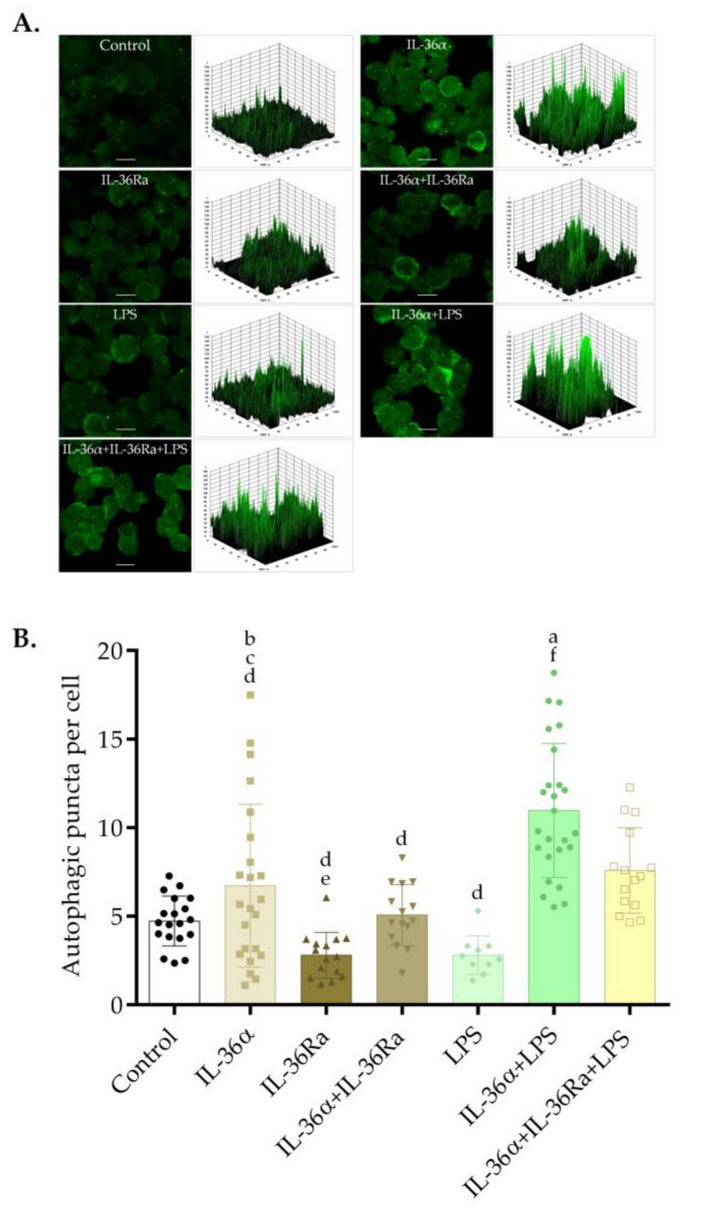
IL-36α and LPS cooperatively increase the accumulation of LC3B-positive vacuoles. THP-1 cells were treated with 10 ng/mL IL-36α, IL-36Ra, and LPS alone or in combination for 6 h, and the intracellular localization of LC3B was analyzed. Control cultures incubated in parallel were left untreated. (**A**) Immunofluorescence assays showing the fluorescence intensities of LC3B-positive vacuoles. The samples were stained for endogenous LC3B protein, and images were obtained by confocal microscopy. The images were subjected to fluorescence intensity analysis by using the Image J software. The 3D surface plots represent the intensity values of the whole image. The results are representative of two independent experiments. Scale bar, 10 µm. (**B**) The average numbers of LC3B-positive autophagic vacuoles. The LC3B-positive autophagic vacuoles were automatically quantified with Image J software. The values on the bar graphs denote the means ± SD of the results of two independent experiments. *p* values were calculated by the ANOVA test with the Tukey post-test. ^a^ *p* < 0.0001 vs. Control; ^b^ *p* < 0.01 vs. IL-36Ra; ^c^ *p* < 0.05 vs. LPS; ^d^ *p* < 0.001 vs. IL-36α‒LPS combination; ^e^ *p* < 0.05 vs. IL-36α‒IL-36Ra‒LPS combination; ^f^ *p* < 0.001 vs. IL-36α‒IL-36Ra‒LPS combination.

Immunofluorescence assays could determine the intracellular localization of LC3B at the 6-h time point and demonstrated that the control cells and the cultures treated with LPS have faint cytoplasmic LC3B staining (Figure 2A). Accordingly, the 3D surface plots revealed a few peaks of low height (Figure 2A). Cells treated with IL-36α displayed staining patterns characterized by faint, punctate LC3B staining (Figure 2A). In contrast, the cells treated with the combination of IL-36α and LPS displayed very bright LC3B staining, and the 3D surface plot consisted of numerous robust peaks (Figure 2A). This result indicates that IL-36α and LPS cooperatively increased the accumulation of LC3B-positive vacuoles.

IL-36 receptor antagonist (IL-36Ra) was used to investigate the role of IL-36α in the synergistic activation of autophagy elicited by the combined treatment with IL-36α and LPS. The cultures were pre-treated with IL-36Ra for 30 min, and then IL-36α or a double combination of IL-36α and LPS were added. The cultures treated either with IL-36Ra alone or in combination with IL-36α displayed a faint cytoplasmic LC3B staining (Figure 2A). Cells treated with the triple combination of IL-36α‒IL-36Ra‒LPS likewise displayed staining patterns characterized by faint, punctate LC3B staining (Figure 2A). Accordingly, the 3D surface plots revealed a few peaks of low height (Figure 2A).

To investigate the effects of IL-36α and LPS on autophagosome formation, the abundances of LC3B-positive vacuoles were determined at the 6-h time point. The average numbers of LC3B-positive vacuoles per cell in the control, IL-36α-, or LPS-treated cultures were 4.72, 6.73, and 2.8, respectively (Figure 2B). Thus, the cells treated with IL-36α alone displayed increased abundances of autophagic vesicles, this difference, however, was not statistically significant. The average numbers of LC3B-positive vacuoles per cell in cultures treated with a combination of IL-36α and LPS were significantly higher than that observed in the control cultures (the average number of autophagosomes in the cultures treated with IL-36α-LPS was 10.97 versus 4.72 in the control, *p* < 0.0001) (Figure 2B). Moreover, the cells treated with the triple combination of IL-36α-IL-36Ra-LPS exhibited significantly lower numbers of LC3B-positive vacuoles per cell than in the cultures treated with the IL-36α-LPS combination: The average number of autophagosomes in the cultures treated with IL-36α-IL-36Ra-LPS was 7.58 vs. 10.97 in cells treated with IL-36α-LPS, *p* < 0.05 (Figure 2B). Thus, although IL-36α and LPS alone do not cause a significant alteration in the number of LC3B-positive vacuoles, the combination of IL-36α and LPS does significantly stimulate the accumulation of autophagosomes.

### 3.2. The Effects of IL-36α and LPS on the Levels of LC3B-I and LC3B-II

**Figure 3:**
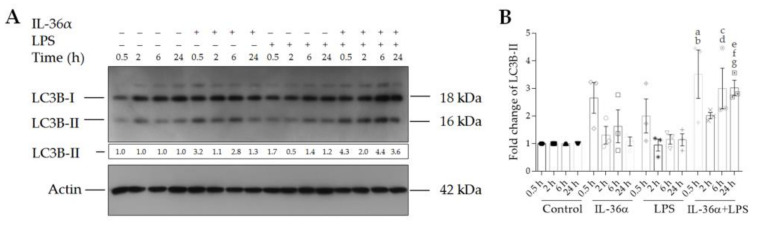
IL-36α and LPS cooperatively increase the level of LC3B-II. (**A**) Western blot analysis showing the kinetics of endogenous LC3B-II expression. Total protein was isolated from controls (lanes 1–4), IL-36α-treated cells (lanes 5–8), LPS-treated cells (lanes 9–12), and cells treated with IL-36α‒LPS combination (lanes 13–16) at the indicated time points. Samples were resolved on SDS-PAGE and transferred to PVDF filters. After incubation with the corresponding antibody, the levels of LC3B-I and LC3B-II were determined with a chemiluminescence detection system. Band intensities were quantified with ImageJ software. The ratios of the protein levels measured at 0.5, 2, 6, and 24 h were compared to the corresponding time point controls and expressed as fold change (shown below each lane). The results are representative of three independent experiments. (**B**) The values on the bar graph denote the means ± SD of the results of three independent experiments. *p* values were calculated by the ANOVA test with the Sidak post-test. ^a^ *p* < 0.01 vs. the 0.5-h time point control, ^b^ *p* < 0.05 vs. the 0.5-h time point LPS, ^c^ *p* < 0.05 vs. the 6-h time point control, ^d^ *p* < 0.01 vs. the 6-h time point LPS, ^e^ *p* < 0.05 vs. the 24-h time point control, ^f^ *p* < 0.01 vs. the 24-h time point IL-36α, and ^g^ *p* < 0.01 vs. the 24-h time point LPS.

The effects of IL-36α and LPS on the levels of LC3B-I and LC3B-II were determined by Western blot analysis (Figure 3 and Figure S1). The control THP-1 cells displayed endogenous expression of both the lipidated and the non-lipidated forms of LC3B at each time point (Figure 3A, lanes 1–4). IL-36α-treated cells exhibited slightly higher LC3B-II levels at the 0.5-, 2-, and 6-h time points than controls (Figure 3A, lanes 5–7). However, these alterations were not statistically significant (Figure 3B). Likewise, there were no significant alterations in LPS-treated cells versus controls (Figure 3). In contrast, the simultaneous treatment of cells with IL-36α and LPS triggered a significant increase in the level of LC3B-II as compared with the controls (at the 0.5-, 6-, and 24-h time points the fold increases of LC3B-II levels in cells treated with IL-36α‒LPS combination were 3.52, *p* < 0.01, 3.0, and 3.02, *p* < 0.05 for both, respectively) (Figure 3A, lanes 13, 15 and 16, and Figure 3B). The LC3B-II level at the 2-h time point dropped compared to the values measured at 0.5, 6, and 24 h after the IL-36α and LPS combined treatment (Figure 3). These data suggest that IL-36α and LPS alone do not increase the level of LC3B-II, whereas combined IL-36α and LPS treatment cooperatively stimulates the lipidation of LC3B with characteristic kinetics.

### 3.3. The Effects of IL-36α and LPS on the Autophagic Flux

**Figure 4:**
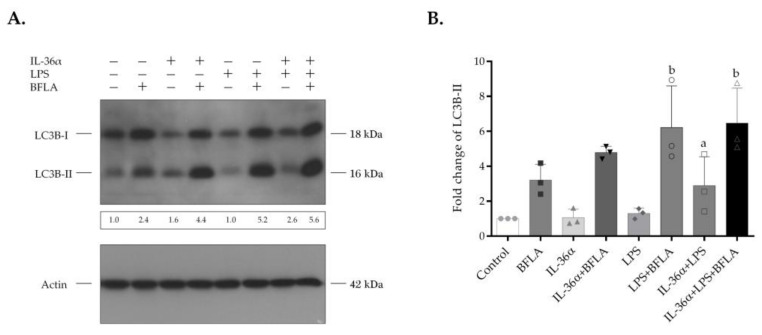
IL-36α and LPS cooperatively stimulate the autophagic flux. THP-1 cells were treated with IL-36α, and LPS alone or in combination for 2 h and then exposed to 100 nM bafilomycin A1 for another 4-h period. The total protein extracted was analyzed for LC3B expression by Western blot analysis. (**A**) Western blot analysis showing increased autophagic flux in cells treated with IL-36α, and LPS alone, or in combination. The ratios of the protein levels were calculated, and expressed as fold change, shown below each lane. Results are representative of three independent experiments. (**B**) The values on the bar graph denote the means ± SD of the results of three independent experiments. *p* values were calculated by the ANOVA test with the Sidak post-test. ^a^ *p* < 0.05 vs. control, ^b^ *p* < 0.05 vs. BFLA control. BFLA, bafilomycin A1.

Bafilomycin A1 (BFLA) is an inhibitor of autophagosome-lysosome fusion and lysosomal hydrolase activity and was used to investigate the autophagic flux (Figure 4 and Figure S2). The cultures were incubated with IL-36α and LPS alone or in combination for 2 h and then treated with BFLA for another 4-h period just before the preparation of cell lysates. Compared with the control, BFLA increased the level of LC3B-II (Figure 4A, lanes 1 and 2, respectively). The elevated LC3B-II level of the BFLA-treated cells indicates that this drug efficiently blocked the autophagic flux under the experimental conditions used. In the presence of BFLA, IL-36α triggered a higher increase in the level of LC3B-II than in the corresponding drug control (Figure 4A, lanes 4 and 2, respectively). However, this alteration was not statistically significant (Figure 4B). In contrast, compared with the BFLA control, LPS—acting singly or in combination with IL-36α—elicited a significant increase in the levels of LC3B-II of cells incubated in the presence of BFLA (the fold increases of LC3B-II levels in cells treated either with LPS alone or the IL-36α‒LPS combination in the presence of BFLA were 6.22, and 6.47, *p* < 0.05 for both) (Figure 4B). In the presence of BFLA, the cells treated with the IL-36α‒LPS combination exhibited higher increases in the level of LC3B-II than cultures stimulated only with LPS. These data indicate that combined IL-36α and LPS treatment cooperatively stimulates the autophagic flux.

### 3.4. The Effects of IL-36α and LPS on the Level of Beclin-1

**Figure 5:**
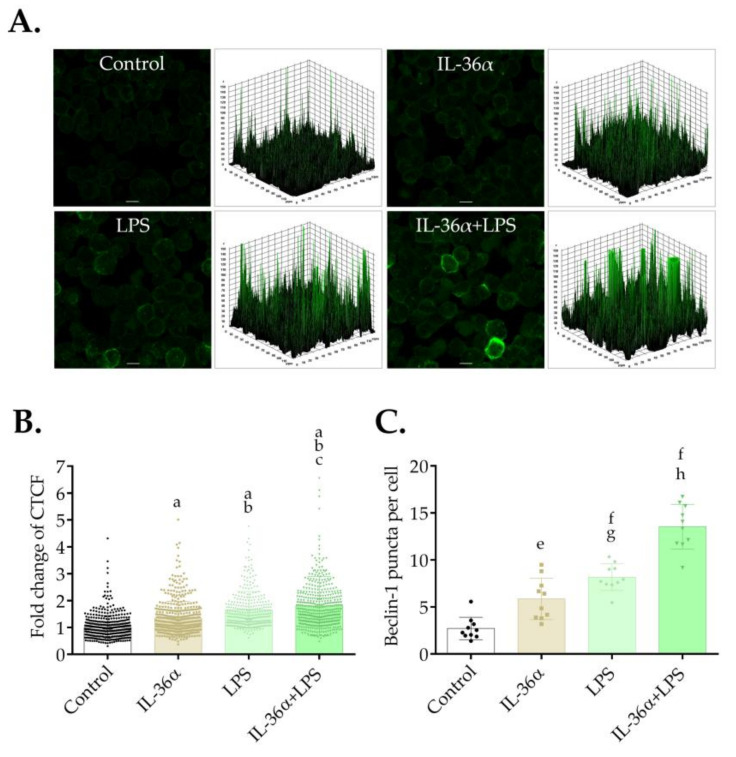
IL-36α and LPS cooperatively increase Beclin-1 levels. THP-1 cells were treated with 10 ng/mL IL-36α, IL-36Ra, and LPS alone or in combination for 6 h, and the level of Beclin-1 fluorescence was analyzed. Control cultures incubated in parallel were left untreated. (**A**) Immunofluorescence assay showing the fluorescence intensities of Beclin-1. The samples were stained for the endogenous Beclin-1 protein, and images were obtained by confocal microscopy. The images were subjected to fluorescence intensity analysis with Image J. The 3D surface plots represent the intensity values of the entire image. The results are representative of two independent experiments. Scale bar, 10 µm. (**B**) Quantification of Beclin-1 fluorescence intensities. The fold changes of CTCF values were calculated as the CTCF of cells treated with 10 ng/mL IL-36α, and LPS alone or in combination/CTCF of control cultures. The values on the bar graphs denote the means ± SD of the results of two independent experiments. *p* values were calculated by the ANOVA test with the Tukey post-test. ^a^ *p* < 0.0001 vs. Control; ^b^ *p* < 0.0001 vs. IL-36α; ^c^ *p* < 0.05 vs. LPS. (**C**) Quantification of the intracellular abundances of Beclin-1 puncta. The Beclin-1-positive puncta were quantified with Image J software. The values on the bar graphs denote the means ± SD of the results of two independent experiments. *p* values were calculated by the ANOVA test with Tukey post-test. ^e^ *p* < 0.01 vs. Control; ^f^ *p* < 0.0001 vs. Control; ^g^ *p* < 0.05 vs. IL-36α; ^h^ *p* < 0.0001 vs. IL-36α and LPS.

The effects of IL-36α and LPS on the level and intracellular localization of Beclin-1 were determined by immunofluorescence assay at the 6-h time point. The control cells showed a faint cytoplasmic Beclin-1 staining (Figure 5A). Accordingly, the 3D surface plots revealed a few peaks of low height (Figure 5A). In contrast, the cells treated with IL-36α and LPS alone or in combination displayed very bright Beclin-1 staining, and the 3D surface plots consisted of numerous robust peaks (Figure 5A). Measurement of the staining intensities showed that IL-36α and LPS acting singly or in combination elicited significant increases as compared with the control (the CTCF values in cells treated with IL-36α, LPS, or IL-36α-LPS combination were 1.35, 1.66, or 1.8 vs. 1.0 in the control, *p* < 0.0001 for all, respectively) (Figure 5B). Measurement of the abundances of Beclin-1-positive vacuoles likewise revealed that IL-36α and LPS acting singly or in combination triggered significant increases as compared with the control (the average numbers of Beclin-1-positive puncta in cells treated with IL-36α, LPS or IL-36α-LPS combination were 5.86, 8.18, or 13.55 vs. 2.72 in the control, *p* < 0.01, *p* < 0.0001 and *p* < 0.0001, respectively) (Figure 5C). These data indicate that IL-36α and LPS cooperatively elevate the level of Beclin-1.

### 3.5. The Effects of IL-36α and LPS on Cellular Signaling in the THP-1 Cell Line

**Figure 6:**
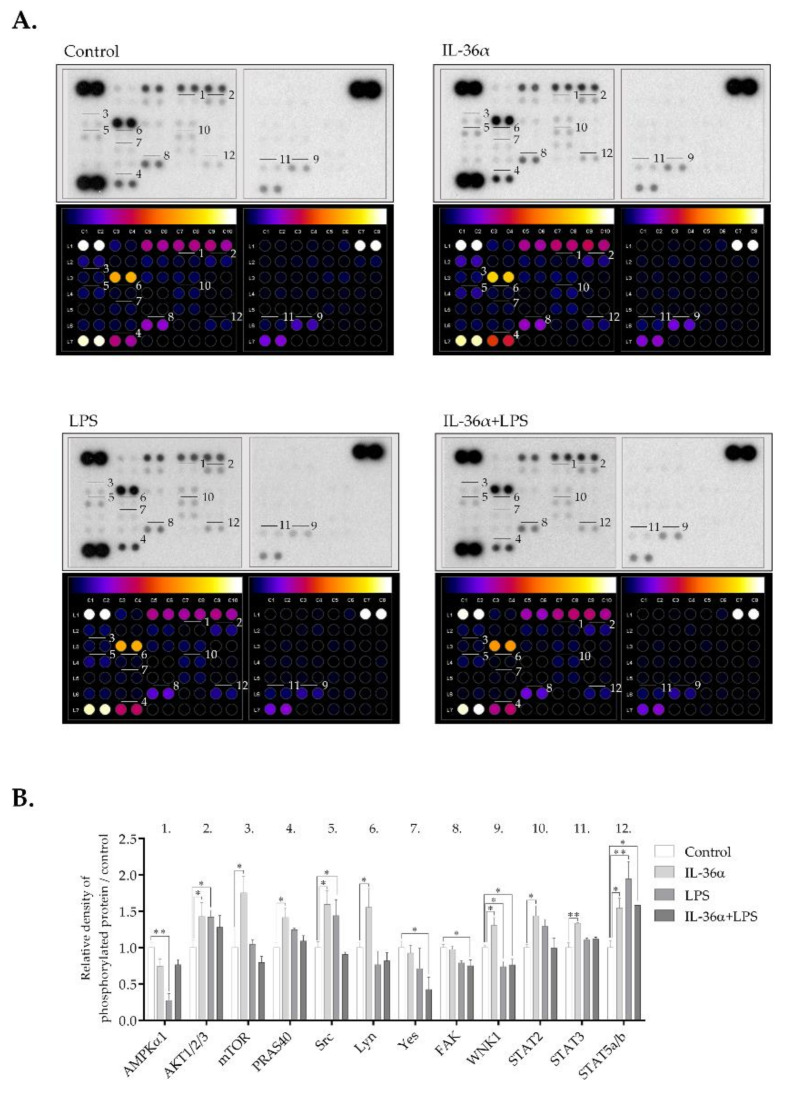
Differential phospho-kinase array profiles of cells treated with IL-36α and LPS. (**A**) Phospho-kinase array analysis. Total protein was isolated from THP-1 cells treated with IL-36α, and LPS alone or in combination for 30 min. Control cells were left untreated. The samples were hybridized with a phospho-kinase array kit. The labeled spots correspond to the phospho-proteins modulated by IL-36α and LPS. (**B**) Quantification of phosphoproteins from the proteomic array (average of duplicate spots). Spot densities of phosphoproteins were quantified using Image J analysis software and normalized to positive controls on the same membrane. *p* values were calculated by the ANOVA test with the Sidak post-test. * *p* < 0.05; ** *p* < 0.01.

A phospho-kinase array that detects the phosphorylation levels of 43 major protein kinases (Table S1) was used to investigate the effect of IL-36α and LPS on the activation level of signaling pathways implicated in autophagy regulation. IL-36α led to the activation of a subset of kinases (Figure 6 and Figure S3). Compared with the control, the most significant effect was an increase in Ak strain transforming factor 1/2/3 (Akt1/2/3) (S473) phosphorylation. The phosphorylation levels of the proline-rich Akt substrate of 40 kDa (PRAS40) (T246) and mechanistic target of rapamycin (mTOR) (S2448)—two signaling molecules downstream of Akt1/2/3)—were also increased (Figure 6 and Figure S3). IL-36α triggered phosphorylation of with no lysine kinase 1 (WNK1) (T60), some steroid receptor coactivator (Src) family kinases including Src (Y419) and Lyn (Y397), and signal transducer and activator of transcription (STAT) family members such as STAT2 (Y689), STAT3 (S727) and STAT5a/b (Y694/Y699). Compared with the control, LPS increased the levels of phospho-Akt1/2/3 (S473), phospho-Src (Y419), and STAT5a/b (Y694/Y699); it decreased phosphorylation of adenosine monophosphate-activated protein kinase α1 (AMPKα1) (Figure 6). Compared with the control, IL-36α and LPS combined treatment increased phosphorylation of STAT5a/b, whereas the levels of phospho-Yes (Y426), phospho-focal adhesion kinase (FAK) (Y397), and phospho-WNK1 (T60) were decreased (Figure 6 and Figure S3). Thus, IL-36α, LPS, and the combined treatment elicit distinct phosphorylation patterns of signaling molecules.

## 4. Discussion

Compelling evidence indicates that cellular autophagic and immune processes are highly intertwined and that their coordinated functioning is essential for the efficient protection of the human body against pathogenic Gram-negative bacteria [33,45,46,47,59]. During infections, cytokines and molecules defined as PAMP act simultaneously to activate partially overlapping signaling pathways. The combined effect may differentially regulate cellular autophagic activity. Thus, this study investigated the impact of IL-36α upon endogenous and LPS-induced autophagy.

To study the autophagic activity of THP-1 cells treated with IL-36α and LPS alone or in combination, we determined the intracellular distribution of LC3B and measured LC3B lipidation as well as the autophagic flux [60]. These experiments demonstrated that the cells treated with IL-36α alone displayed increased abundances of autophagic vesicles, elevated endogenous LC3B-II levels, and stimulated autophagic flux; these differences, however, were not statistically significant (Figure 2, Figure 3 and Figure 4). Recent observations indicated that IL-36β and IL-36γ activate the autophagic process in primary murine CD4^+^CD25^+^ Treg cells [57] and human macrophages [56], respectively. There may be several explanations for the weaker pro-autophagic effect of IL-36α revealed in our present study such as differences between IL-36 subtypes and different sensitivities of various cell types to this cytokine. Consistent with previous findings [52,53,61], our results demonstrated that LPS significantly increased autophagosome synthesis (Figure 3). The combination of IL-36α and LPS raised the intensity level of LC3B staining and cooperatively stimulated the translocation of this protein into autophagic vesicles (Figure 2). Supporting this observation, we found that IL-36Ra significantly inhibited the pro-autophagic effect of the IL-36α/LPS combined treatment (Figure 2). The IL-36α‒LPS combination elevated LC3B-II and decreased LC3B-I levels indicating that the lipidation of LC3B is highly stimulated (Figure 3). Finally, our experiments showed that in cultures treated with the combination of IL-36α and LPS, the autophagic flux is increased considerably by this cytokine/PAMP combination (Figure 4). These results suggest that IL-36α and LPS cooperatively stimulate autophagy.

To investigate the effect of IL-36α and LPS on the activation of some signaling pathways, we determined the phosphorylation levels of protein kinases implicated in autophagy regulation (Figure 6). Our studies have shown that IL-36α increased the phosphorylation of Akt1/2/3 (S473), PRAS40 (T246), mTOR (S2448), WNK1 (T60), and some Src as well as STAT family kinases such as Src, Lyn, STAT2, STAT3, and STAT5a/b. PRAS40 is a negative regulator of mTORC1 [34,62]. Akt- and mTORC1-mediated phosphorylation of PRAS40 results in its dissociation from mTORC1 that in turn alleviates inhibition of mTORC1 and blocks induction of the autophagic cascade [34,62]. An interesting recent study revealed that IL-36β stimulates mTORC1 via the PI3K/Akt, IκB kinase and MyD88 pathways [63]. Our experiments demonstrate that, like IL-36β, IL36α activates mTORC1 via the PIK/Akt pathway. Moreover, we suggest that the Akt-mediated activation of mTOR involves PRAS40. Other previous studies indicated that WNK1 acts as an autophagy inhibitor by interfering with the activation of AMPK and PI3KC3 [64]. STAT3 has been shown to regulate autophagy in localization- and context-dependent manners and can elicit both pro-autophagic and anti-autophagic effects [65]. In light of these observations, our data suggest that in the early phase of IL-36α signaling, both anti- and pro-autophagic pathways are activated. Our observations show that LPS increases phosphorylation of Akt1/2/3, Src as well as STAT5a/b and decreases the level of phospho-AMPKα1 in THP-1 cells; this is fully consistent with previous reports [66,67]. Interestingly, the phosphorylation pattern of cells incubated with IL36α and LPS differed from the signatures detected either in IL36α- or LPS-treated cells. We found that the IL36α/LPS combined treatment increased phosphorylation of STAT5a/b, had minimal effect on the Akt/PRAS40/mTOR pathway, and reduced the levels of phospho-Yes, phospho-FAK, and phospho-WNK1. Thus, the combined treatment of IL-36α and LPS appears to dampen PI3K/Akt/mTOR, FAK and WNK1 signaling. The TLR4 signal transduction network is known to be kept under strict control by multiple mechanisms including positive and negative crosstalk regulations that maintain the integrity of immune cells by preventing excessive inflammation [68]. The negative regulators acting through the activation of transcription play a primary role in the late phase of TLR and IL-36 signaling; their role in the early stage thus can be excluded. Important studies, however, revealed that PI3K has an essential role in the safety mechanism controlling the early-stage of TLR4-mediated signaling [69]. PI3K can suppress TLR4 signaling by altering the availability of phosphatidylinositol-(4,5)bisphosphate (PIP_2_) at the cytoplasmic membrane and hence can modulate the intracellular localization of adaptors, the magnitude of activation, and signal output [70]. Based on these observations, we suggest that the combinatorial effect of IL-36α/LPS may exert an excessive PI3K activation, which—while not suspending the inhibition of downstream events of autophagy—can significantly reduce it by decreasing the Akt-mediated activation of mTORC1.

Previous studies have demonstrated that Beclin-1 plays a pivotal role in the autophagic process. Beclin-1 was shown to interact with Bcl-2 and Bcl-X_L_, which suppresses autophagosome biogenesis. The release of Beclin-1 is a prerequisite for the formation of a functional PI3KC3 complex. Phosphorylation events and TRAF6-mediated ubiquitination of Beclin-1 may destabilize Beclin-1‒Bcl-2/Bcl-X_L_ association and abrogate BECN1-BCL2/Bcl-X_L_ interaction. Some IL-36α and LPS signaling intermediates have the potential to regulate the functional activity of Beclin-1 [71,72]. Thus, we investigated the effect of IL-36α and LPS on Beclin-1 protein. Our results showed that IL-36α and LPS acting singly or in combination elevated the staining intensities and increased the abundances of Beclin-1-positive vacuoles (Figure 5). The IL-36α/LPS treatment was again more efficient than IL-36α or LPS alone. These data further support the notion that IL-36α and LPS cooperatively promote the autophagic process because increased Beclin-1 levels were shown to correlate with enhanced autophagy [60].

Our results suggest a hypothetical model for the mechanism of the enhanced pro-autophagic effect observed in cells treated with IL-36α and LPS simultaneously. The IL-36α/LPS combination reduces the activation level of the PI3K/Akt/mTORC1 axis by triggering rapid depletion of PIP_2_ at the cytoplasmic membrane. As a result, mTOR-mediated inhibition of autophagy is alleviated. Some components of the IL-36α and LPS signaling networks are known to induce autophagy. MyD88 binds directly whereas TRAF3 and TRAF6 ubiquitinate the Beclin-1 protein, thus disrupting the interaction between Beclin-1 and Bcl-2 [52,53]. This results in increased oligomerization of Beclin-1, activation of the PI3KC3 complex, and initiation of autophagosome formation [52,53]. The IL-36α/LPS combination increases the activation level of PI3KC3 complex directly and subsequently stimulates autophagy. Thus, this cytokine/PAMP combination triggers pro-autophagic biased signaling by several mechanisms and thereby cooperatively stimulates the autophagic cascade (Figure S4). Previous studies have shown that bacteria affect the autophagic cascade and, conversely, autophagy influences the infection process. The bacteria studied so far all interact with the autophagic machinery but in different ways. The structural components, PAMPs, and exotoxins of several bacteria induce autophagy. However, some bacteria can effectively prevent autophagic recognition, inhibit autophagy initiation and maturation of autophagosomes or block the fusion of lysosomes with autophagosomes, while others hijack the autophagic compartment to support their intracellular survival. Our data indicate that cytokines may modify the pro-autophagic effect of bacterial PAMPs. An increased xenophagic activity of innate immune cells exposed to IL-36α and LPS—functioning as part of the cell-autonomous defense system—may play a protective role in the pathogenesis of infections caused by Gram-negative bacteria [73,74]. Our data indicate that cytokines may modify the pro-autophagic effect of bacterial PAMPs. An increased xenophagic activity of innate immune cells exposed to IL-36α and LPS—functioning as part of the cell-autonomous defense system—may play an important role in the pathogenesis of infections caused by Gram-negative bacteria.

In conclusion, our results demonstrate that the IL-36α/LPS triggers pro-autophagic biased signaling by several mechanisms and thereby stimulates the autophagic cascade cooperatively in the THP-1 cell line. However, some limitations of this investigation, such as the need for additional experimental evidence that can corroborate the synergistic effect of this cytokine/PAMP combination in other cell types, have yet to be addressed. Moreover, further studies are needed to investigate the effect of increased autophagic activity on the functions of innate immune cells treated with IL-36α and LPS simultaneously.

## Data Availability

Data presented in this study are available on request from the corresponding author.

## Supplementary Materials

The following are available online at https://www.mdpi.com/article/10.3390/biomedicines9111541/s1, Figure S1: IL-36α and LPS cooperatively increase the level of LC3B-II., Figure S2: IL-36α and LPS cooperatively stimulate the autophagic flux. Figure S3: Differential phospho-kinase array profiles of cells treated with IL-36α and LPS. Figure S4: IL36α and LPS cooperatively induced autophagy by multiple mechanisms. Table S1: List of the detected phospho-proteins by using the Proteome ProfilerTM human phospho-kinase array kit.

## References

[1] Dinarello C.A. (2018). Overview of the IL-1 Family in Innate Inflammation and Acquired Immunity. Immunol. Rev..

[2] Garlanda C., Dinarello C.A., Mantovani A. (2013). The Interleukin-1 Family: Back to the Future. Immunity.

[3] Murrieta-Coxca J.M., Rodríguez-Martínez S., Cancino-Diaz M.E., Markert U.R., Favaro R.R., Morales-Prieto D.M. (2019). IL-36 Cytokines: Regulators of Inflammatory Responses and Their Emerging Role in Immunology of Reproduction. Int. J. Mol. Sci..

[4] Smith D.E., Renshaw B.R., Ketchem R.R., Kubin M., Garka K.E., Sims J.E. (2000). Four New Members Expand the Interleukin-1 Superfamily. J. Biol. Chem..

[5] Clancy D.M., Henry C.M., Sullivan G.P., Martin S.J. (2017). Neutrophil Extracellular Traps Can Serve as Platforms for Processing and Activation of IL-1 Family Cytokines. FEBS J..

[6] Henry C.M., Sullivan G.P., Clancy D.M., Afonina I.S., Kulms D., Martin S.J. (2016). Neutrophil-Derived Proteases Escalate Inflammation through Activation of IL-36 Family Cytokines. Cell Rep..

[7] Zhou L., Todorovic V., Atassi M.Z. (2021). Interleukin-36: Structure, Signaling and Function. Protein Reviews: Volume 21.

[8] Towne J.E., Garka K.E., Renshaw B.R., Virca G.D., Sims J.E. (2004). Interleukin (IL)-1F6, IL-1F8, and IL-1F9 Signal through IL-1Rrp2 and IL-1RAcP to Activate the Pathway Leading to NF-KappaB and MAPKs. J. Biol. Chem..

[9] Boraschi D., Tagliabue A. (2013). The Interleukin-1 Receptor Family. Semin. Immunol..

[10] Yi G., Ybe J.A., Saha S.S., Caviness G., Raymond E., Ganesan R., Mbow M.L., Kao C.C. (2016). Structural and Functional Attributes of the Interleukin-36 Receptor. J. Biol. Chem..

[11] Bridgewood C., Stacey M., Alase A., Lagos D., Graham A., Wittmann M. (2017). IL-36γ Has Proinflammatory Effects on Human Endothelial Cells. Exp. Dermatol..

[12] Foster A.M., Baliwag J., Chen C.S., Guzman A.M., Stoll S.W., Gudjonsson J.E., Ward N.L., Johnston A. (2014). IL-36 Promotes Myeloid Cell Infiltration, Activation, and Inflammatory Activity in Skin. J. Immunol..

[13] Vigne S., Palmer G., Martin P., Lamacchia C., Strebel D., Rodriguez E., Olleros M.L., Vesin D., Garcia I., Ronchi F. (2012). IL-36 Signaling Amplifies Th1 Responses by Enhancing Proliferation and Th1 Polarization of Naive CD4+ T Cells. Blood.

[14] Johnston A., Xing X., Guzman A.M., Riblett M., Loyd C.M., Ward N.L., Wohn C., Prens E.P., Wang F., Maier L.E. (2011). IL-1F5, -F6, -F8, and -F9: A Novel IL-1 Family Signaling System That Is Active in Psoriasis and Promotes Keratinocyte Antimicrobial Peptide Expression. J. Immunol..

[15] Qu Q., Zhai Z., Xu J., Li S., Chen C., Lu B. (2020). IL36 Cooperates with Anti-CTLA-4 MAbs to Facilitate Antitumor Immune Responses. Front. Immunol..

[16] Harusato A., Abo H., Ngo V.L., Yi S.W., Mitsutake K., Osuka S., Kohlmeier J.E., Li J.D., Gewirtz A.T., Nusrat A. (2017). IL-36γ Signaling Controls the Induced Regulatory T Cell–Th9 Cell Balance via NFκB Activation and STAT Transcription Factors. Mucosal. Immunol..

[17] Ngo V.L., Kuczma M., Maxim E., Denning T.L. (2021). IL-36 Cytokines and Gut Immunity. Immunology.

[18] Bassoy E.Y., Towne J.E., Gabay C. (2018). Regulation and Function of Interleukin-36 Cytokines. Immunol. Rev..

[19] Gresnigt M.S., Rösler B., Jacobs C.W.M., Becker K.L., Joosten L.A.B., van der Meer J.W.M., Netea M.G., Dinarello C.A., van de Veerdonk F.L. (2013). The IL-36 Receptor Pathway Regulates Aspergillus Fumigatus-Induced Th1 and Th17 Responses. Eur. J. Immunol..

[20] Mazgaeen L., Gurung P. (2020). Recent Advances in Lipopolysaccharide Recognition Systems. IJMS.

[21] Steimle A., Autenrieth I.B., Frick J.-S. (2016). Structure and Function: Lipid A Modifications in Commensals and Pathogens. Int. J. Med. Microbiol..

[22] Brubaker S.W., Bonham K.S., Zanoni I., Kagan J.C. (2015). Innate Immune Pattern Recognition: A Cell Biological Perspective. Annu. Rev. Immunol..

[23] Kieser K.J., Kagan J.C. (2017). Multi-Receptor Detection of Individual Bacterial Products by the Innate Immune System. Nat. Rev. Immunol..

[24] Ciesielska A., Matyjek M., Kwiatkowska K. (2021). TLR4 and CD14 Trafficking and Its Influence on LPS-Induced pro-Inflammatory Signaling. Cell. Mol. Life Sci..

[25] Gay N.J., Symmons M.F., Gangloff M., Bryant C.E. (2014). Assembly and Localization of Toll-like Receptor Signalling Complexes. Nat. Rev. Immunol..

[26] Boonen B., Alpizar Y.A., Meseguer V.M., Talavera K. (2018). TRP Channels as Sensors of Bacterial Endotoxins. Toxins.

[27] Schappe M.S., Szteyn K., Stremska M.E., Mendu S.K., Downs T.K., Seegren P.V., Mahoney M.A., Dixit S., Krupa J.K., Stipes E.J. (2018). Chanzyme TRPM7 Mediates the Ca^2+^ Influx Essential for Lipopolysaccharide-Induced Toll-Like Receptor 4 Endocytosis and Macrophage Activation. Immunity.

[28] Scheraga R.G., Abraham S., Niese K.A., Southern B.D., Grove L.M., Hite R.D., McDonald C., Hamilton T.A., Olman M.A. (2016). TRPV4 Mechanosensitive Ion Channel Regulates Lipopolysaccharide-Stimulated Macrophage Phagocytosis. J. Immunol..

[29] Shi J., Zhao Y., Wang Y., Gao W., Ding J., Li P., Hu L., Shao F. (2014). Inflammatory Caspases Are Innate Immune Receptors for Intracellular LPS. Nature.

[30] Dikic I., Elazar Z. (2018). Mechanism and Medical Implications of Mammalian Autophagy. Nat. Rev. Mol. Cell. Biol..

[31] Yang Z., Klionsky D.J. (2010). Mammalian Autophagy: Core Molecular Machinery and Signaling Regulation. Curr. Opin. Cell. Biol..

[32] Parzych K.R., Klionsky D.J. (2014). An Overview of Autophagy: Morphology, Mechanism, and Regulation. Antioxid. Redox Signal..

[33] Khandia R., Dadar M., Munjal A., Dhama K., Karthik K., Tiwari R., Yatoo M.I., Iqbal H.M.N., Singh K.P., Joshi S.K. (2019). A Comprehensive Review of Autophagy and Its Various Roles in Infectious, Non-Infectious, and Lifestyle Diseases: Current Knowledge and Prospects for Disease Prevention, Novel Drug Design, and Therapy. Cells.

[34] Sarkar S. (2013). Regulation of Autophagy by MTOR-Dependent and MTOR-Independent Pathways: Autophagy Dysfunction in Neurodegenerative Diseases and Therapeutic Application of Autophagy Enhancers. Biochem. Soc. Trans..

[35] Kim Y.C., Guan K.-L. (2015). MTOR: A Pharmacologic Target for Autophagy Regulation. J. Clin. Invest..

[36] Kim J., Guan K.-L. (2019). MTOR as a Central Hub of Nutrient Signalling and Cell Growth. Nat. Cell Biol..

[37] Yang J., Carra S., Zhu W.-G., Kampinga H.H. (2013). The Regulation of the Autophagic Network and Its Implications for Human Disease. Int. J. Biol. Sci..

[38] Carlsson S.R., Simonsen A. (2015). Membrane Dynamics in Autophagosome Biogenesis. J. Cell Sci..

[39] Lamb C.A., Yoshimori T., Tooze S.A. (2013). The Autophagosome: Origins Unknown, Biogenesis Complex. Nat. Rev. Mol. Cell Biol..

[40] Ichimura Y., Kirisako T., Takao T., Satomi Y., Shimonishi Y., Ishihara N., Mizushima N., Tanida I., Kominami E., Ohsumi M. (2000). A Ubiquitin-like System Mediates Protein Lipidation. Nature.

[41] Mizushima N. (2020). The ATG Conjugation Systems in Autophagy. Curr. Opin. Cell Biol..

[42] Mizushima N., Noda T., Yoshimori T., Tanaka Y., Ishii T., George M.D., Klionsky D.J., Ohsumi M., Ohsumi Y. (1998). A Protein Conjugation System Essential for Autophagy. Nature.

[43] Klionsky D.J., Schulman B.A. (2014). Dynamic Regulation of Macroautophagy by Distinctive Ubiquitin-like Proteins. Nat. Struct. Mol. Biol..

[44] Nakamura S., Yoshimori T. (2017). New Insights into Autophagosome–Lysosome Fusion. J. Cell Sci..

[45] Levine B., Mizushima N., Virgin H.W. (2011). Autophagy in Immunity and Inflammation. Nature.

[46] Sharma V., Verma S., Seranova E., Sarkar S., Kumar D. (2018). Selective Autophagy and Xenophagy in Infection and Disease. Front. Cell Dev. Biol..

[47] Deretic V. (2012). Autophagy: An Emerging Immunological Paradigm. J. Immunol..

[48] Qian M., Fang X., Wang X. (2017). Autophagy and Inflammation. Clin. Transl. Med..

[49] Ge Y., Huang M., Yao Y. (2018). Autophagy and Proinflammatory Cytokines: Interactions and Clinical Implications. Cytokine Growth Factor Rev..

[50] Weichhart T., Hengstschläger M., Linke M. (2015). Regulation of Innate Immune Cell Function by MTOR. Nat. Rev. Immunol..

[51] Katholnig K., Linke M., Pham H., Hengstschläger M., Weichhart T. (2013). Immune Responses of Macrophages and Dendritic Cells Regulated by MTOR Signalling. Biochem. Soc. Trans..

[52] Shi C.-S., Kehrl J.H. (2008). MyD88 and Trif Target Beclin 1 to Trigger Autophagy in Macrophages. J. Biol. Chem..

[53] Shi C.S., Kehrl J.H. (2010). TRAF6 and A20 Regulate Lysine 63-Linked Ubiquitination of Beclin-1 to Control TLR4-Induced Autophagy. Sci. Signal..

[54] Harris J. (2011). Autophagy and Cytokines. Cytokine.

[55] Orosz L., Papanicolaou E.G., Seprényi G., Megyeri K. (2016). IL-17A and IL-17F Induce Autophagy in RAW 264.7 Macrophages. Biomed. Pharmacother..

[56] Gao Y., Wen Q., Hu S., Zhou X., Xiong W., Du X., Zhang L., Fu Y., Yang J., Zhou C. (2019). IL-36γ Promotes Killing of Mycobacterium Tuberculosis by Macrophages via WNT5A-Induced Noncanonical WNT Signaling. J. Immunol..

[57] Ge Y., Huang M., Dong N., Yao Y.-M. (2020). Effect of Interleukin-36β on Activating Autophagy of CD4+CD25+ Regulatory T Cells and Its Immune Regulation in Sepsis. J. Infect. Dis..

[58] Schneider C.A., Rasband W.S., Eliceiri K.W. (2012). NIH Image to ImageJ: 25 Years of Image Analysis. Nat. Methods..

[59] Gomes L.C., Dikic I. (2014). Autophagy in Antimicrobial Immunity. Mol. Cell..

[60] Klionsky D.J., Abdel-Aziz A.K., Abdelfatah S., Abdellatif M., Abdoli A., Abel S., Abeliovich H., Abildgaard M.H., Abudu Y.P., Acevedo-Arozena A. (2021). Guidelines for the Use and Interpretation of Assays for Monitoring Autophagy (4th Edition)^1^. Autophagy.

[61] Delgado M.A., Elmaoued R.A., Davis A.S., Kyei G., Deretic V. (2008). Toll-like Receptors Control Autophagy. EMBO J..

[62] Nascimento E.B.M., Ouwens D.M. (2009). PRAS40: Target or Modulator of MTORC1 Signalling and Insulin Action?. Arch. Physiol. Biochem..

[63] Zhao X., Chen X., Shen X., Tang P., Chen C., Zhu Q., Li M., Xia R., Yang X., Feng C. (2019). IL-36β Promotes CD8+ T Cell Activation and Antitumor Immune Responses by Activating MTORC1. Front. Immunol..

[64] Gallolu Kankanamalage S., Lee A.-Y., Wichaidit C., Lorente-Rodriguez A., Shah A.M., Stippec S., Whitehurst A.W., Cobb M.H. (2016). Multistep Regulation of Autophagy by WNK1. Proc. Natl. Acad. Sci. USA.

[65] You L., Wang Z., Li H., Shou J., Jing Z., Xie J., Sui X., Pan H., Han W. (2015). The Role of STAT3 in Autophagy. Autophagy.

[66] Fan K., Lin L., Ai Q., Wan J., Dai J., Liu G., Tang L., Yang Y., Ge P., Jiang R. (2018). Lipopolysaccharide-Induced Dephosphorylation of AMPK-Activated Protein Kinase Potentiates Inflammatory Injury via Repression of ULK1-Dependent Autophagy. Front. Immunol..

[67] Rex J., Albrecht U., Ehlting C., Thomas M., Zanger U.M., Sawodny O., Häussinger D., Ederer M., Feuer R., Bode J.G. (2016). Model-Based Characterization of Inflammatory Gene Expression Patterns of Activated Macrophages. PLoS Comput. Biol..

[68] Oda K., Kitano H. (2006). A Comprehensive Map of the Toll-like Receptor Signaling Network. Mol. Syst. Biol..

[69] Fukao T., Koyasu S. (2003). PI3K and Negative Regulation of TLR Signaling. Trends Immunol..

[70] Aksoy E., Taboubi S., Torres D., Delbauve S., Hachani A., Whitehead M.A., Pearce W.P., Berenjeno I.M., Nock G., Filloux A. (2012). The P110δ Isoform of the Kinase PI(3)K Controls the Subcellular Compartmentalization of TLR4 Signaling and Protects from Endotoxic Shock. Nat. Immunol..

[71] Boutouja F., Brinkmeier R., Mastalski T., El Magraoui F., Platta H.W. (2017). Regulation of the Tumor-Suppressor BECLIN 1 by Distinct Ubiquitination Cascades. Int. J. Mol. Sci..

[72] Xu H.-D., Qin Z.-H., Qin Z.-H. (2019). Beclin 1, Bcl-2 and Autophagy. Autophagy: Biology and Diseases.

[73] Huang J., Brumell J.H. (2014). Bacteria-Autophagy Interplay: A Battle for Survival. Nat. Rev. Microbiol..

[74] Wu Y.-W., Li F. (2019). Bacterial Interaction with Host Autophagy. Virulence.

