# Supplementary material for: IL-36α and Lipopolysaccharide Cooperatively Induce Autophagy by Triggering Pro-Autophagic Biased Signaling
Source: Biomedicines. 2021 Oct 26;9(11):1541. doi: 10.3390/biomedicines9111541 (PMC8615041; doi:10.3390/biomedicines9111541)
Supplement: Supplementary file 1 [file biomedicines-09-01541-s001.zip › biomedicines-1415053-supplementary/Supplementary Materials-2021-10-23.pdf]

Supplementary Materials

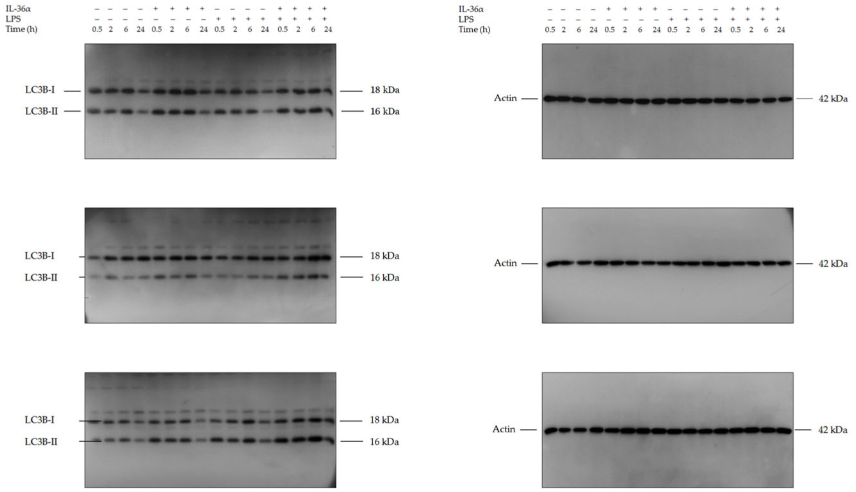

Figure S1. IL-36α and LPS cooperatively increase the level of LC3B-II.

Western blot images used to calculate the fold changes of LC3B-II shown in Figure 2B.

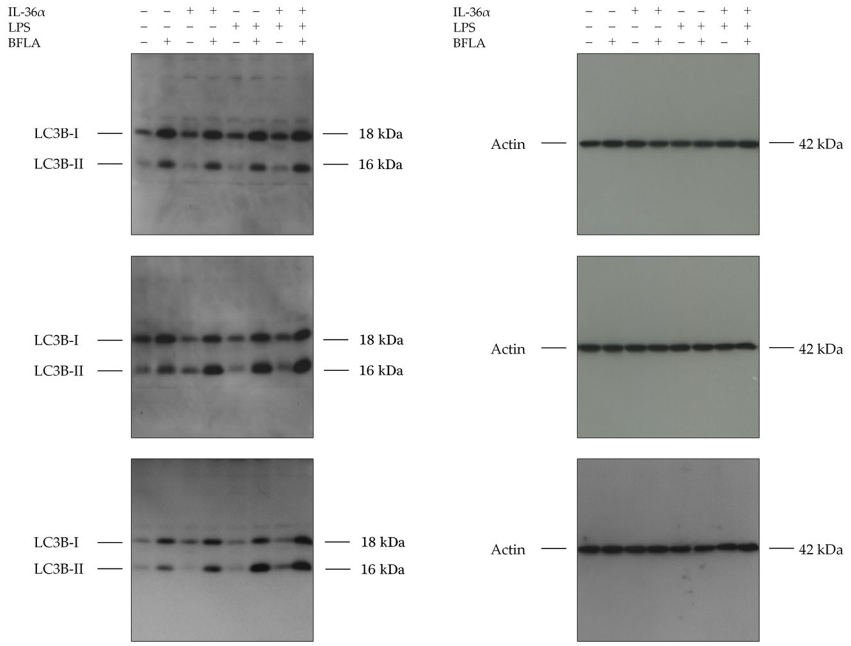

Figure S2. IL-36α and LPS cooperatively stimulate the autophagic flux.

Western blot images used to calculate the fold changes of LC3B-II shown in Figure 3B.

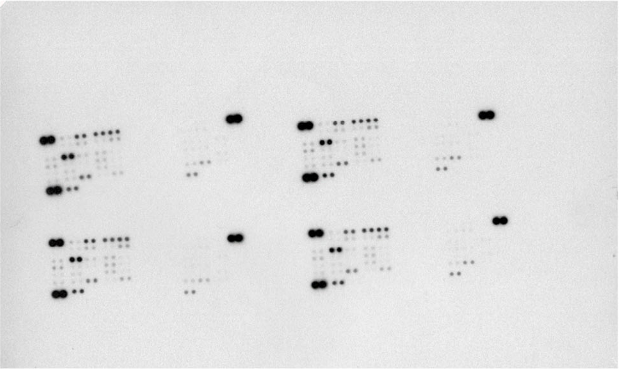

Figure S3. Differential phospho-kinase array profiles of cells treated with IL-36α and LPS.

Uncropped image of the phospho-kinase array used to create Figure 5.

Table S1. List of the detected phospho-proteins by using the Proteome Profiler™ human phospho-kinase array kit.

Membrane/ Coordinate	Target/Control	Phosphorylation Site
**A-A1, A2**	Reference Spot	-
**A-A3, A4**	p38α	T180/Y182
**A-A5, A6**	ERK1/2	T202/Y204, T185/ Y187
**A-A7, A8**	JNK1/2/3	T183/Y185, T221/ Y223
**A-A9, A10**	GSK-3α/β	S21/S9
**B-A13, A14**	p53	S392
**B-A17, A18**	Reference Spot	-
**A-B3, B4**	EGFR	Y1086
**A-B5, B6**	MSK1/2	S376/S360
**A-B7, B8**	AMPKα1	T183
**A-B9, B10**	Akt 1/2/3	S473
**B-B11, B12**	Akt 1/2/3	T308
**B-B13, B14**	p53	S46
**A-C1, C2**	TOR	S2448
**A-C3, C4**	CREB	S133
**A-C5, C6**	HSP27	S78/S82
**A-C7, C8**	AMPKα2	T172
**A-C9, C10**	β-Catenin	-
**B-C11, C12**	p70 S6 Kinase	T389
**B-C13, C14**	p53	S15
**B-C15, C16**	c-Jun	S63
**A-D1, D2**	Src	Y419
**A-D3, D4**	Lyn	Y397
**A-D5, D6**	Lck	Y394
**A-D7, D8**	STAT2	Y689
**A-D9, D10**	STAT5a	Y694
**B-D11, D12**	p70 S6 Kinase	T421/S424
**B-D13, D14**	RSK1/2/3	S380/S386/S377
**B-D15, D16**	eNOS	S1177
**A-E1, E2**	Fyn	Y420
**A-E3, E4**	Yes	Y426
**A-E5, E6**	Fgr	Y412
**A-E7, E8**	STAT6	Y641
**A-E9, E10**	STAT5b	Y699
**B-E11, E12**	STAT3	Y705
**B-E13, E14**	p27	T198
**B-E15, E16**	PLC-γ1	Y783
**A-F1, F2**	Hck	Y411
**A-F3, F4**	Chk-2	T68
**A-F5, F6**	FAK	Y397
**A-F7, F8**	PDGF Rβ	Y751
**A-F9, F10**	STAT5a/b	Y694/Y699
**B-F11, F12**	STAT3	S727
**B-F13, F14**	WNK1	T60
**B-F15, F16**	PYK2	Y402
**A-G1, G2**	Reference Spot	-

**A-G3, G4**	PRAS40	T246
**A-G9, G10**	PBS (Negative Control)	-
**B-G11, G12**	HSP60	-
**B-G17, G18**	PBS (Negative Control)	-

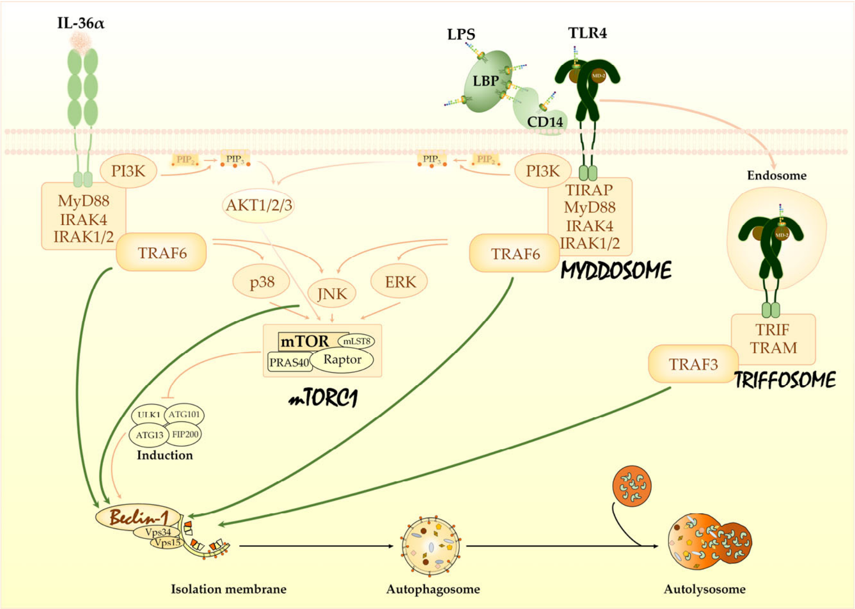

Figure S4. IL36α and LPS cooperatively induced autophagy by multiple mechanisms.

The IL-36α/LPS combination reduces the activation level of the PI3K/Akt/mTORC1 axis by triggering rapid depletion of PIP2 at the cytoplasmic membrane. As a result, mTOR-mediated inhibition of autophagy is alleviated. Additionally, the IL-36α/LPS combination increases the activation level of PI3KC3 complex via the activation of MyD88, TRAF3, and TRAF6. As a result, autophagosome formation is stimulated. Thus, this cytokine/PAMP combination triggers pro-autophagic biased signaling by several mechanisms and thereby stimulates the autophagic cascade cooperatively.
